# Wearable IoT-Enabled Galvanic Skin Response Device for Objective Pain and Stress Monitoring: Hardware Design and Prototype Development

**DOI:** 10.3390/s26010116

**Published:** 2025-12-24

**Authors:** Anushka N. Phadke, Khawlah Harasheh, Satinder Gill

**Affiliations:** 1 Department of Biomedical Engineering, Virginia Commonwealth University, Richmond, VA 23298, USA; phadkean@vcu.edu; 2 Department of Information Systems, J. Sargeant Reynolds Community College, Richmond, VA 23228, USA

**Keywords:** assistive technologies, visually impaired, haptics, admittance control, dead reckoning, tactile displays

## Abstract

Accurate pain and stress assessment remains a challenge in patients with limited communication ability. Current galvanic skin response (GSR) devices lack real-time feedback, wireless communication, and robustness against motion artifacts, limiting their clinical utility. This paper presents the design and development of a wearable internet-of-things (IoT) enabled GSR system incorporating Bluetooth Low Energy (BLE) communication, ergonomic mechanical housing, and artifact-filtering through a custom API. The system integrates finger-mounted electrodes, a custom amplifier and signal processor, an nRF52840 BLE microcontroller, and a rechargeable Li-ion battery in a compact 3D-printed wrist-mounted enclosure. Basic validation with two healthy subjects demonstrated reliable detection of stress-induced GSR fluctuations with reduced movement artifacts. Results indicate the feasibility of the proposed design as a low-cost, wireless, and ergonomic solution for objective pain and stress monitoring.

## 1. Introduction

Pain is one of the most common reasons people seek medical care, yet its assessment remains a major challenge in healthcare. Current clinical practice often relies on subjective methods such as self-report scales or clinician observation of behavioral cues. While useful, these approaches have well-known limitations. Self-report requires the patient to be conscious, communicative, and cognitively able to describe their pain, which is not possible for infants, people with cognitive impairments, sedated patients, or those with limited language ability. Observational tools, such as behavioral pain scales, are also prone to subjectivity and variability. The result is that pain is frequently underdiagnosed or mischaracterized, which can delay treatment and worsen outcomes. These limitations have motivated growing interest in objective physiological measures of pain [1,2,3,4,5]. 

One such measure is electrodermal activity (EDA), also known as Galvanic Skin Response (GSR). EDA reflects changes in skin conductance caused by eccrine sweat gland activity, which is regulated by the sympathetic branch of the autonomic nervous system. Since sympathetic activity increases with stress, arousal, and nociception, EDA provides a non-invasive, real-time signal that can serve as an indirect marker of pain. EDA is measured by placing electrodes on high-density sweat gland areas such as the fingers or palm, applying a small voltage, and recording skin conductance in microsiemens (µS). Because the measurement is simple, non-invasive, and low cost, EDA has been studied extensively as a potential biomarker of pain and related states [1,2,3].

Multiple studies have shown that EDA responds consistently to painful stimulation. Posada-Quintero and Chon [6,7,8] validated EDA responses during controlled thermal pain experiments, showing reliable increases in conductance during nociceptive events [3,4,5,6]. Bhatkar et al. [9] combined EDA with timing of peak pain in a cold pressor test to quantify sympathetic activation. Hamilton et al. [7] extended this work to movement-related nociception, showing that EDA sensors can detect changes in pain during dynamic activities. Walas et al. [10] applied skin conductance measures in infants undergoing procedures, demonstrating that EDA can provide useful information when behavioral assessment alone is insufficient. Kong et al. [11] developed a wrist-worn, smartphone-linked EDA system for real-time acute pain detection, illustrating the feasibility of mobile deployment.

Beyond raw signal use, EDA has been integrated into computational frameworks for pain detection [2,12,13]. Susam et al. [2] used machine learning models trained on EDA data to automatically classify pain states, showing that combining physiological signals with data-driven methods can improve accuracy. These studies together highlight that EDA is sensitive to pain across populations and contexts. However, many implementations remain limited to laboratory settings or specialized hardware. Devices are often tethered, lack wireless communication, or are not designed for continuous use in everyday life.

There is a need for wearable, low-cost, and easy-to-use systems that can monitor pain in everyday life [14,15]. This follows a broader trend in healthcare technology. Populations worldwide are aging, and chronic health conditions such as musculoskeletal pain and neuropathy are becoming more common. At the same time, healthcare costs are rising unsustainably. Continuous monitoring solutions that extend beyond clinics could help address these challenges by enabling early detection of problems, guiding interventions, and supporting self-management.

The success of wearable systems in related domains highlights their potential impact. In studies of gait and balance, wearable accelerometers and gyroscopes have been adopted as portable alternatives to motion capture or force plate systems [16,17,18,19]. These devices enable continuous monitoring of mobility in everyday environments and provide more detailed information than occasional clinic visits. Research on assistive technology for blind and visually impaired users has also shown the importance of creating systems that are low-cost, portable, and usable in daily life [3,4]. Collectively, these areas demonstrate how portability and accessibility can make advanced sensing systems practical outside laboratory settings. The same principle applies to pain monitoring: devices must be wearable, wireless, and unobtrusive.

The development of Bluetooth Low Energy (BLE) has been a key factor in enabling wearable health monitoring. BLE allows low-power wireless data transmission, which is well suited to battery-powered devices. BLE uses less power than classic Bluetooth while still providing adequate range and data transfer for physiological monitoring [20]. Systematic reviews and surveys have shown its wide use in remote health monitoring systems, rehabilitation, and consumer wearables [21,22]. BLE has also been extended to mesh networks, which can support larger-scale monitoring applications [22]. Early work by Omre and Keeping [23] demonstrated the potential of Bluetooth connectivity in medical devices, while more recent efforts highlight the role of BLE in internet-of-things (IoT) based healthcare monitoring frameworks [24]. These developments make it possible to design compact, wireless systems that can connect seamlessly to smartphones or cloud platforms.

For GSR-based pain monitoring, BLE enables continuous, near real-time transmission of data to mobile devices for storage, analysis, and feedback. This opens the door to integration with electronic health records, telemedicine platforms, and machine learning models for automated pain assessment. Portability and wireless connectivity also allow patients to use the device in natural environments, improving ecological validity compared to laboratory-based assessments.

While technical feasibility is necessary, usability often determines whether wearable systems are adopted in practice. Studies of gait monitoring systems have shown that accuracy alone is insufficient; devices must also be unobtrusive, reliable, and easy for older adults or patients with limited technical knowledge to use [16,25]. Similarly, research on tactile displays for blind and visually impaired individuals highlights the need for designs that do not introduce unnecessary complexity or cognitive load [3,4]. These lessons are directly relevant to pain monitoring. If devices are bulky, uncomfortable, or require complicated setup, patient compliance will be low, and clinical trust will be limited. A successful system must therefore combine technical accuracy with user-centered design.

In this paper, we describe the design and implementation of a compact, wireless GSR monitoring device for pain assessment. The system uses an off-the-shelf GSR sensor with the XIAO nRF52840 Sense microcontroller. It collects GSR signals, processes them in real time, and transmits the data using BLE. A custom strap and enclosure ensure stable skin contact, and filtering algorithms reduce noise and motion artifacts. The device also supports communication with mobile applications through an API for feedback and scalable data handling. This system is intended as a step toward practical, IoT-enabled pain monitoring in clinical and everyday environments.

## 2. Materials and Methods

### 2.1. System Overview

**Figure 1:**
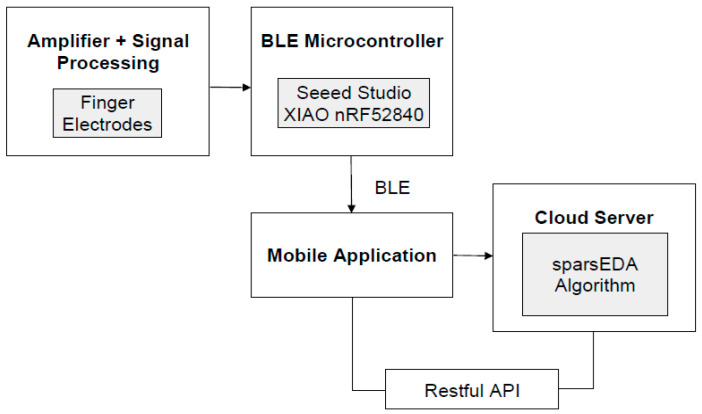
Block diagram illustrating the interaction between functional blocks of the system.

The wearable GSR system consists of four primary subsystems: sensing, amplification & signal processing, communication, and power supply. The overall system architecture and interaction between these functional blocks is shown in Figure 1.

**Figure 2:**
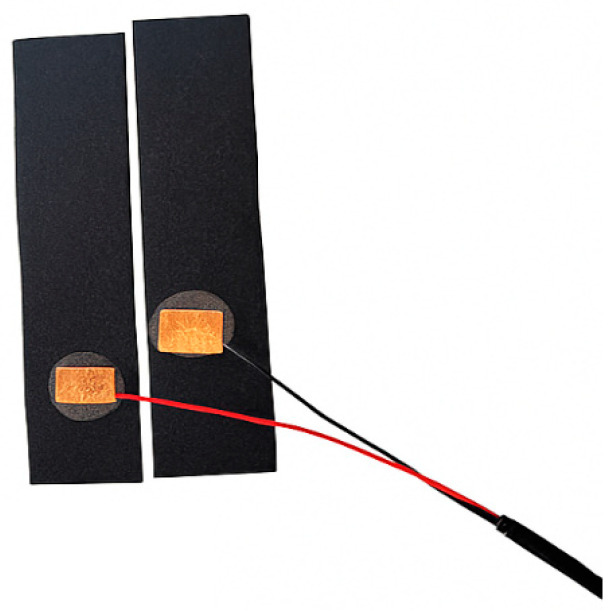
Dry electrodes fabricated using conductive tape and integrated into Velcro-mounted fingertip sleeves.

The sensing stage uses non-gelled dry contact electrodes placed on the index and middle fingers to measure changes in skin resistance [26]. The electrodes are fabricated using conductive-tape and embedded into Velcro-mounted fingertip sleeves to ensure stable skin contact and adaptability across different users. The physical layout and electrode design is shown in Figure 2.

The amplification and signal processing module measures the change in resistance and use voltage divider to convert changes into resistance into voltage signal suitable for digital conversion.

**Figure 3:**
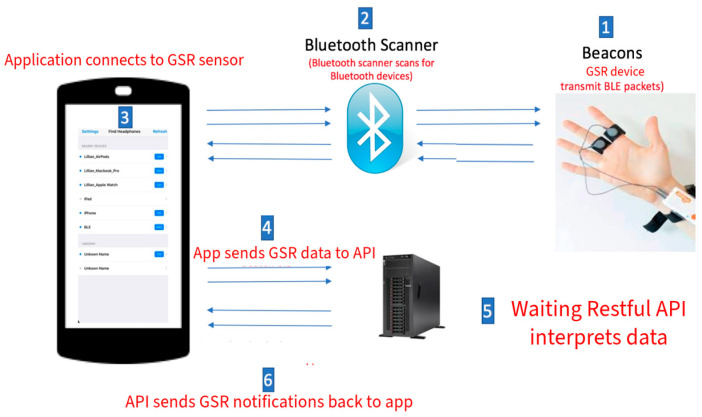
End-to-end data flow of GSR measurements from sensor to mobile app via BLE and API. (1) GSR electrodes acquire physiological signals from the user; (2) the Bluetooth scanner detects available BLE devices; (3) the mobile application establishes a connection with the GSR device; (4) the mobile application transmits GSR data to the backend API; (5) the RESTful API processes and interprets the received data; and (6) the API sends processed GSR notifications back to the mobile application.

The communication module is built around the Seeed Studio XIAO nRF52840 Sense microcontroller (Seeed Studio, Shenzhen, China), a compact Bluetooth Low Energy (BLE) board optimized for low-power wireless applications. The BLE stack enables short-range communication with a custom mobile application. As shown in Figure 3, data from the microcontroller is transmitted to the mobile platform and relayed via a RESTful API to a server running the sparsEDA algorithm, which filters noise and isolates stress-related electrodermal activity events in near real time.

**Figure 4:**
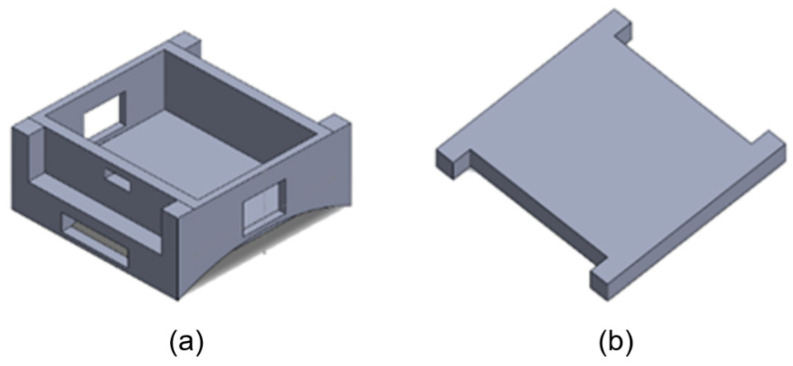
CAD visualization of the device enclosure: (**a**) base enclosure and (**b**) lid component.

**Figure 5:**
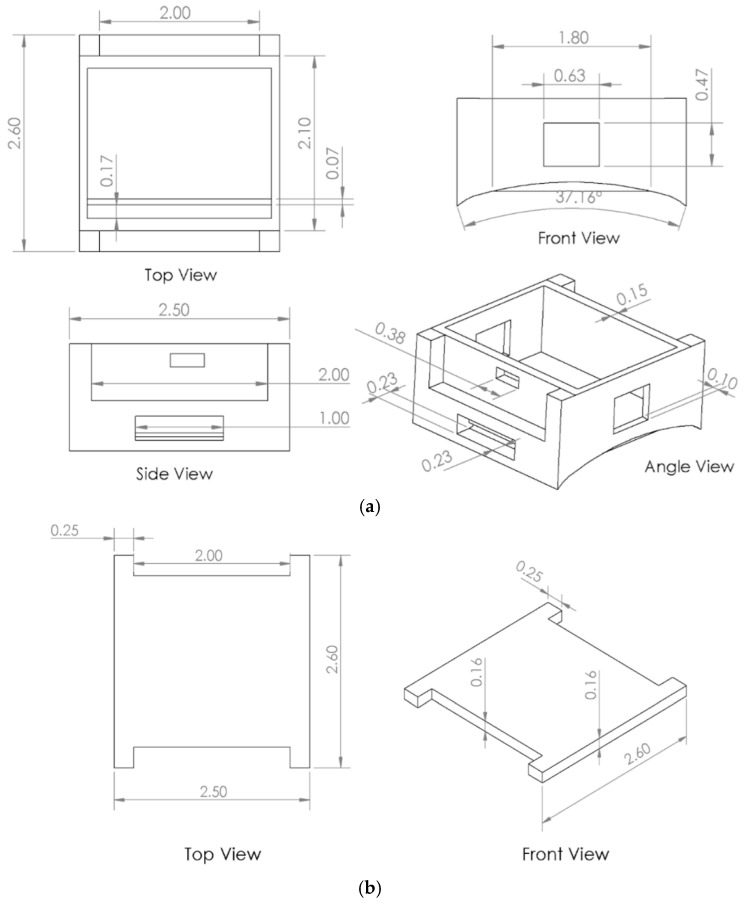
Engineering drawings of the device base (**a**) and lid (**b**), showing orthographic and isometric views with dimensions.

The power subsystem employs a rechargeable 3.7 V lithium-ion battery connected through an onboard charging circuit. The hardware is enclosed in a 3D-printed wrist-mounted housing, featuring dedicated compartments for the microcontroller and battery, as well as slots for Velcro straps to provide ergonomic fit and stability. The 3D design of the enclosure is shown in Figure 4, and the detailed engineering drawings of the base and lid are shown in Figure 5a,b respectively, with all dimensions reported in inches. 

**Figure 6:**
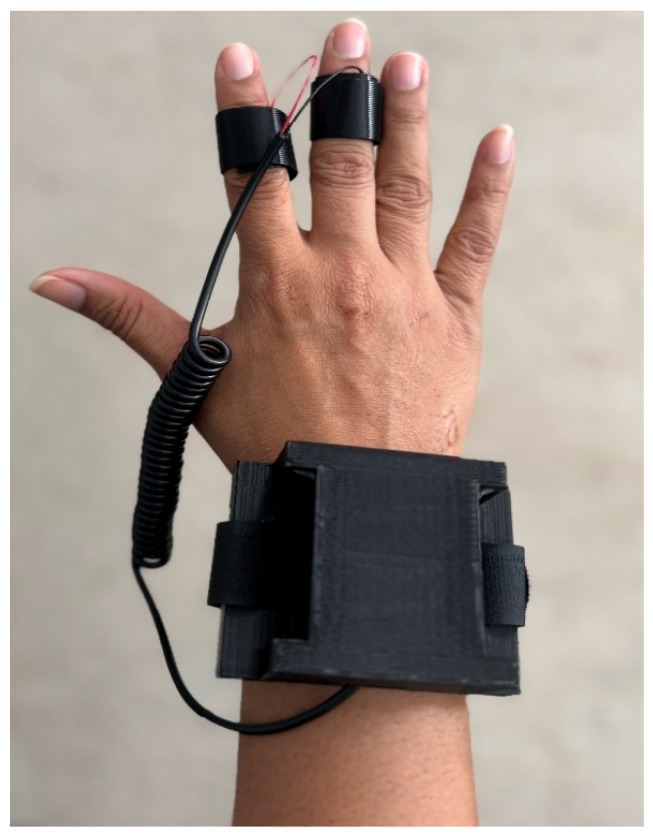
Volunteer wearing the GSR device.

Finally, Figure 6 shows the completed GSR device worn by volunteers. This modular design not only ensures comfort and durability during prolonged use but also simplifies assembly and maintenance. Together, these subsystems create an affordable, wireless, and ergonomic platform for real-time stress and pain monitoring.

### 2.2. Amplification & Signal Processing

The amplification and signal processing stage is necessary because the raw voltage output from the skin–electrode interface is too small and unstable for direct use. Skin resistance can vary widely, typically from 50 kΩ to over 500 kΩ, and when placed in a voltage divider this produces only small voltage fluctuations, often in the millivolt range. The relationship can be described by the standard divider equation:

(1) $$V_{out}=\frac{V_{vcc}\times R_{skin}}{R_{ref}+R_{skin}}$$

where *R_skin_* is the resistance of the skin–electrode interface, *R_ref_* is the reference resistor in the voltage divider, *V_cc_* and is the supply voltage. Because these variations are so small, they are easily masked by noise and fall below the resolution of the microcontroller’s analog-to-digital converter (ADC). An intermediate conditioning stage is therefore required to bias, amplify, and filter the signal before digitization [27]. 

**Figure 7:**
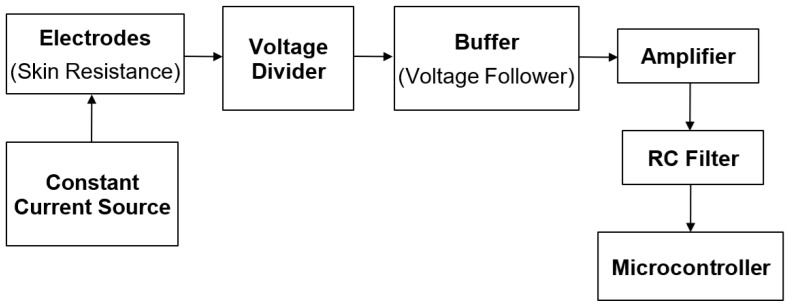
GSR Signal Conditioning Architecture.

As shown in Figure 7, the circuit is designed to convert variations in skin resistance into a measurable voltage signal for electrodermal activity analysis. A constant current source excites the skin through a pair of electrodes, generating a voltage that changes proportionally with skin conductance. This signal is first scaled by a voltage divider network to maintain it within the operational range of the subsequent stages.

The buffered stage, configured as a voltage follower, electrically isolates the electrodes from the amplifier to prevent loading and ensure accurate signal transfer. The amplifier stage then applies controlled gain to enhance sensitivity to subtle fluctuations in skin resistance. Finally, an RC filter smooths transient noise and high-frequency components, providing a stable, conditioned analog output suitable for acquisition by the microcontroller’s analog-to-digital converter (ADC).

By combining voltage division, buffering, amplification, and filtering in this sequence, the circuit transforms subtle changes in skin resistance into a clean analog voltage that reliably reflects electrodermal activity. This conditioned signal is then routed to the microcontroller for digitization and subsequent analysis.

### 2.3. Main Processor

The main processor is a commercially available Seeed Studio XIAO nRF52840 Sense microcontroller, which integrates Nordic Semiconductor’s nRF52840 system-on-chip. The board provides built-in Bluetooth Low Energy (BLE) connectivity, onboard sensors, and support for a variety of peripherals, making it well suited for compact, wearable systems.

Within this device, the microcontroller serves as the central control unit. It digitizes analog inputs from the electrodes using its 12-bit analog-to-digital converter (ADC), organizes the data into structured packets, and manages wireless transmission through the integrated BLE stack. These capabilities enable reliable short-range communication with the mobile application while maintaining low energy consumption.

A key characteristic of the nRF52840 is its power efficiency. The chip supports multiple low-power operating modes and efficient duty cycling of the BLE radio, which extends battery life when paired with a small lithium-ion cell. The combination of processing capability, integrated wireless communication, and energy efficiency makes this microcontroller an ideal choice for a wearable pain and stress monitoring system.

### 2.4. SparsEDA Algorithm 

Electrodermal activity (EDA) signals are inherently noisy, combining both slow tonic variations and fast phasic responses that correspond to stress or pain events. Traditional analysis methods often struggle to distinguish between meaningful physiological responses and motion artifacts or baseline drift, which is a critical limitation in wearable systems. To address this, our system employs the sparsEDA algorithm, a sparse deconvolution method specifically designed for EDA signal analysis [25].

**Figure 8:**
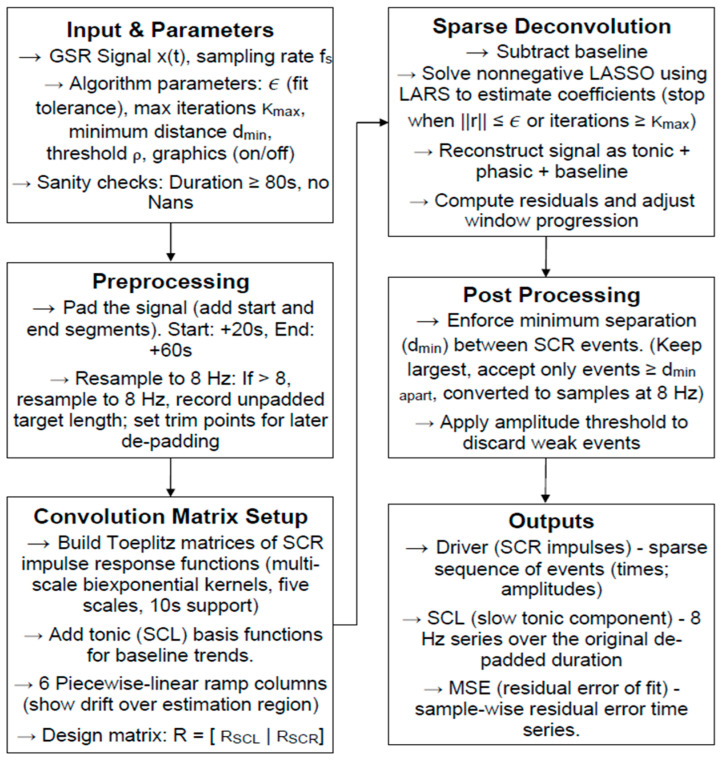
sparsEDA Flowchart.

The core principle behind sparsEDA is that true skin conductance responses (SCRs) are sparse in time—that is, they occur only at specific moments when the sympathetic nervous system is activated. The algorithm models the observed EDA signal as a combination of: (a) a tonic component (slow-changing baseline skin conductance), (b) a sparse phasic component (discrete SCR peaks corresponding to stress or pain events), and (c) noise and artifacts from movement, electrode shifts, or environmental conditions. Figure 8 illustrates the complete processing pipeline of the sparsEDA algorithm, including preprocessing, sparce deconvolution, post-processing, and extraction of tonic and phasic components. 

Using convex optimization techniques, sparsEDA decomposes the raw EDA signal into these three components, isolating the sparse SCR events of interest. This approach provides both the timing and amplitude of physiological responses with high temporal resolution, while filtering out unrelated fluctuations. Compared to simple thresholding or moving average methods, sparsEDA is significantly more robust to motion and environmental noise, making it particularly well suited for wearable applications.

In our implementation, the algorithm was deployed on a cloud-based server to handle real-time data streams. Raw GSR data collected by the wearable device is transmitted via Bluetooth Low Energy (BLE) to the mobile app, which then forwards it through a RESTful API to the server. The server executes sparsEDA on incoming signals, performs near real-time artifact removal, and returns processed data back to the app with minimal latency. Running the algorithm in the cloud, rather than on the microcontroller, allowed us to leverage higher computational power for fast deconvolution while keeping the wearable lightweight and energy-efficient. This architecture ensures that the device can deliver reliable, real-time feedback without compromising usability or battery life.

A key step in sparsEDA is the way it represents each SCR. Rather than treating changes in conductance as arbitrary fluctuations, the algorithm assumes that SCRs follow a stereotyped shape, with a rapid onset and a slower recovery. By fitting this shape to the signal, sparsEDA can locate the exact start of each response and measure its length. This event-based representation provides more detailed information than the raw signal. Instead of a continuous trace, the output is a sequence of discrete responses, each with a defined onset time and amplitude. These can be counted, averaged, or aligned with external events, making them easier to interpret and more useful for comparing between conditions. From these outputs, features such as the frequency of responses, their average size, or their timing relative to painful stimuli can be extracted. These features form a more reliable basis for analysis to detect pain.

**Figure 9:**
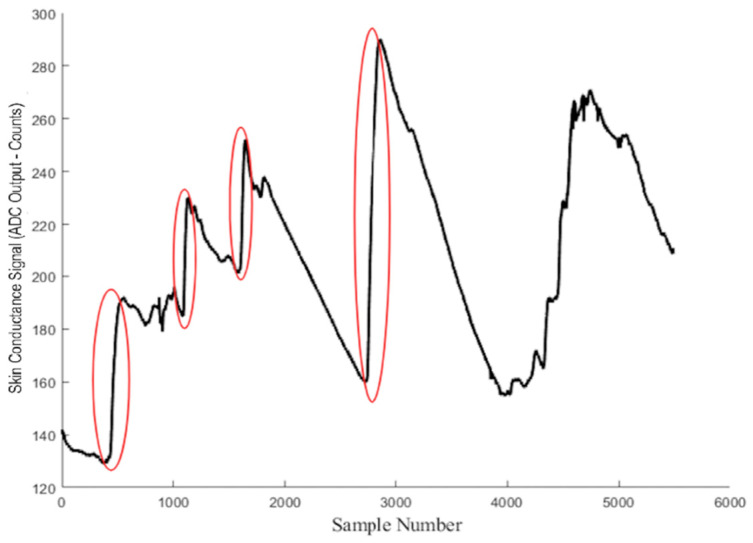
Representative GSR signal expressed as raw analog-to-digital (ADC) output (counts) versus sample number.

When a person experiences stress or pain, the sympathetic nervous system becomes more active. This leads to increased activity in sweat glands on the fingers and palms, which changes the skin’s electrical conductance. These changes appear as SCRs with a rapid rise and slower recovery. The number and size of these SCRs provide an objective way to measure how strongly someone reacts to stress or painful events. By detecting and quantifying these responses using the sparsEDA processing pipeline (Figure 9), it becomes possible to assess stress and pain in a consistent manner that does not rely solely on self-report or observation.

## 3. Results

Results are presented to validate the ability of the GSR device to measure skin conductance levels in response to different events. Two volunteers were tested using the system. Each person wore the GSR device on their fingers, and their eyes were covered to limit visual distractions. Stress stimuli were introduced by randomly clapping near the participant’s ear. The timing of each clapping event was recorded using a timer and these time points were used to verify alignment between the stimuli and the observed rapid increases in the skin conductance signal. These sharp rises correspond to stimulus-related skin conductance responses rather than unrelated signal fluctuations.

The collected signals were analyzed using the sparsEDA algorithm. This algorithm helped separate the slow changes in skin conductance from the fast changes caused by sudden events. By doing this, it was possible to see both the overall trend and the specific responses to each event.

**Figure 10:**
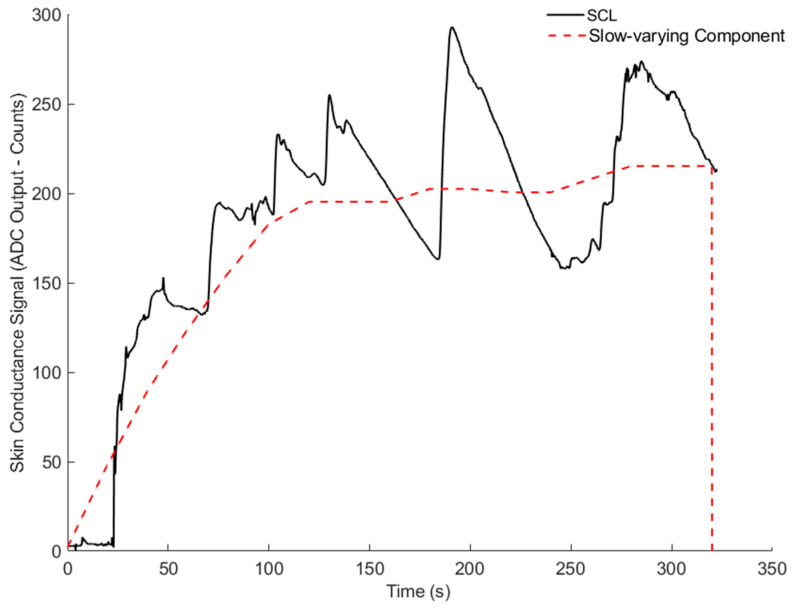
Skin conductance signal (ADC output - counts) and slow-varying component for Volunteer 1.

**Figure 11:**
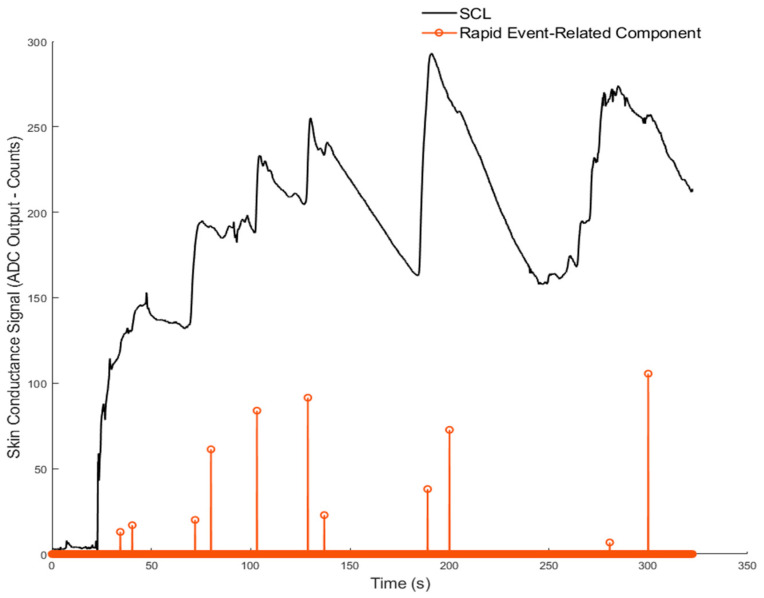
Event-related skin conductance responses detected by the sparsEDA algorithm from ADC-based GSR signals for Volunteer 1.

**Figure 12:**
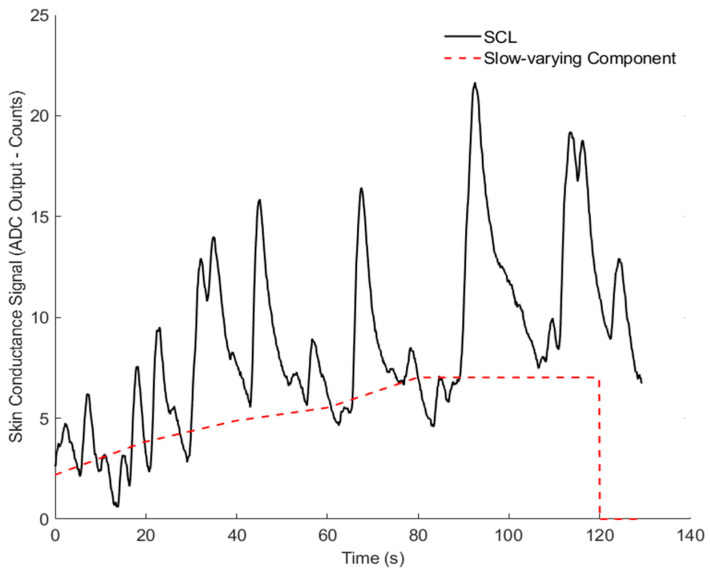
Skin conductance signal (ADC output-counts) and slow-varying component for Volunteer 2.

**Figure 13:**
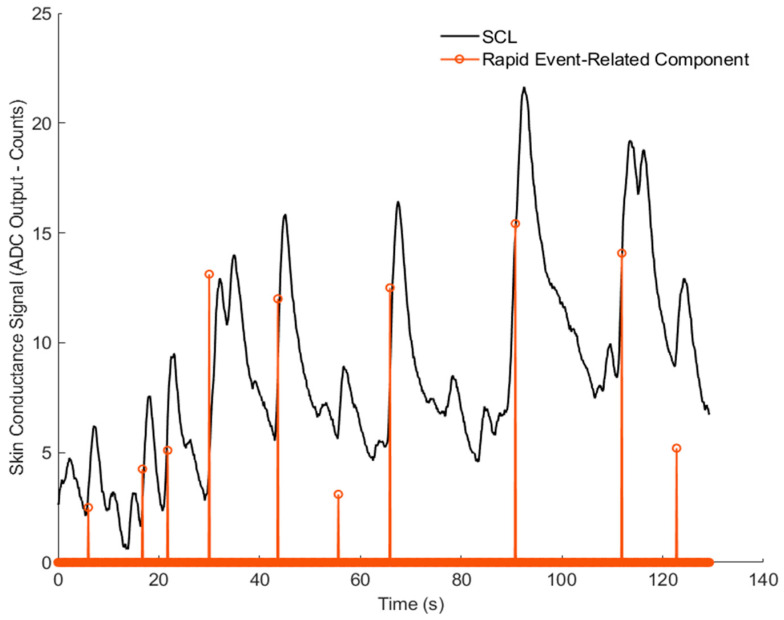
Event-related skin conductance responses detected by the sparsEDA algorithm from ADC-based GSR signals for Volunteer 2.

Figure 10 and Figure 11 show the results for Volunteer 1. Figure 10 presents the slow, baseline changes, while Figure 11 highlights the rapid changes related to the stress events. Figure 12 and Figure 13 show similar results for Volunteer 2, with clear differences between the slow and fast components of the signal.

These results suggest that the GSR device can successfully collect reliable data, and the sparsEDA algorithm can detect event-related responses in the skin conductance signal.

## 4. Discussion 

This paper presents the design and implementation of a wearable, wireless GSR monitoring system that integrates sensing hardware, Bluetooth Low Energy (BLE) communication, and cloud-based signal processing for near real-time identification of stress-related events. The proposed architecture demonstrates how lightweight wearable electronics can be combined with scalable computational resources to produce a practical platform for pain and stress monitoring. By leveraging the sparsEDA algorithm in the cloud, the system is able to isolate stress-related skin conductance responses while minimizing the computational burden on the microcontroller. This division of tasks allows the wearable to remain compact and power-efficient while still delivering advanced signal analysis with low latency.

The key contribution of this work is not large-scale validation, but rather the demonstration of a feasible system architecture that addresses several limitations of current GSR devices. Through ergonomic mechanical design, stable electrode placement, wireless data transmission, and near real-time cloud-based processing, the system shows that it can successfully collect data, process signals, and identify stress-related responses. These results provide an important foundation for further development toward a fully deployable wearable solution.

Future work will focus on advancing both the device design and the security architecture. On the hardware side, the next stage will involve reducing the device footprint by transitioning from an off-the-shelf microcontroller to a custom printed circuit board (PCB). This change will improve wearability, reduce power consumption, and allow for tighter system integration, ultimately making the device more practical for long-term use. Another major step will be to evaluate the system in structured, real-world, stress-inducing environments to better understand how it performs under natural conditions. These studies will also support subject-specific calibration. Baseline skin conductance will be recorded under resting conditions, and relative changes will be measured during controlled stress tasks. This approach will help relate conductance changes to stress or pain levels while reducing differences between subjects. This evaluation was beyond the scope of the present design-focused study but is an important direction for future investigation. The research team is currently engaged in the Institutional Review Board (IRB) approval process to enable human-subject studies, which will be essential for moving the system from a prototype stage to a clinically validated and field-ready tool for everyday use.

In parallel, future work will also focus on strengthening end-to-end security while maintaining the lightweight and efficient design required for IoT devices. Because the system connects to multiple environments to share, save, and analyze data, it is critical that all communication and storage layers remain secure without overwhelming the device’s limited resources. Traditional security mechanisms are often too heavy for low-power IoT systems, so our approach will use lightweight algorithms and technologies guided by the STRIDE framework to ensure strong protection from data capture to transmission and cloud storage. STRIDE is a model that helps identify and address six main types of security threats: Spoofing, Tampering, Repudiation, Information Disclosure, Denial of Service, and Elevation of Privilege. We are developing five complementary security shields to achieve this balance. The first shield focuses on wireless and Bluetooth security, protecting the device from eavesdropping, man-in-the-middle, and replay attacks through Bluetooth Low Energy (LE) Secure Connections with authenticated pairing (such as Quick Response (QR) or Near Field Communication (NFC)–based out-of-band methods), encrypted and authenticated Generic Attribute Profile (GATT) operations, private address rotation, and rate-limiting to prevent repeated connection attempts The second shield secures communication between the device and authorized mobile applications by binding each unit to a verified account using mutual Transport Layer Security (mTLS) and certificate pinning, with all credentials stored in an encrypted operating system keystore and short-lived tokens issued with least-privilege access.

The third shield focuses on firmware integrity, supply chain hardening, and over-the-air (OTA) updates. It will implement secure boot with signed firmware validation to prevent unauthorized modifications, and anti-rollback protection using dual A/B update slots that automatically verify system health and revert to a safe version if errors are detected. Each device will also receive a unique cryptographic identity during manufacturing through secure key injection, and all debug interfaces (Serial Wire Debug/Joint Test Action Group) will be locked to prevent hardware probing or cloning. The fourth shield targets cloud and API security by enforcing TLS 1.3 with mTLS for two-way authentication, routing all traffic through an API gateway with rate limiting and token validation to prevent denial-of-service attacks. All sensitive information will remain encrypted under key management or hardware security modules (Key Management System/Hardware Security Module), with audit logs maintained for traceability and data integrity checks supported by counters and timestamps. Finally, the fifth shield focuses on physical hardware protection, where cryptographic keys will be stored in secure enclaves such as TrustZone-M, automatically wiped after abnormal activity or failed authentication attempts. Additional defenses like biometric or impedance-based authentication will be explored to prevent spoofing of physiological signals.

Across all five shields, STRIDE threat modeling will guide risk identification, mitigation, and verification testing to ensure comprehensive protection. Heavy computational security tasks will be performed in the cloud, while only lightweight operations will run locally on the device to preserve processing efficiency. Together, these hardware, software, and security advancements will move the system closer to a compact, reliable, and trustworthy tool ready for both clinical validation and real-world deployment.

## 5. Conclusions

This paper proposes the design of a wearable, wireless GSR monitoring system that integrates ergonomic hardware, BLE communication, and cloud-based signal processing for near real-time identification of stress-related electrodermal activity. The system demonstrates that it is feasible to collect data, transmit it wirelessly, and process it using the sparsEDA algorithm with low latency. While the current work focuses on design and feasibility, further development is ongoing. 

## Data Availability

The original contributions presented in this study are included in the article. Further inquiries can be directed to the corresponding author.
